# Acute appendicitis within a femoral hernia (De Garengeot hernia): A case report

**DOI:** 10.1016/j.radcr.2026.10.038

**Published:** 2026-11-05

**Authors:** Rubi Chander, Jarvis Pereira, Misbahuddin Ansari

**Affiliations:** Department of Radiology, South West Acute Hospital, Western Health and Social Care Trust, Enniskillen, Northern Ireland, United Kingdom

**Keywords:** De Garengeot hernia, Femoral hernia, Acute appendicitis, Computed tomography, Groin mass

## Abstract

De Garengeot hernia is a rare form of femoral hernia in which the vermiform appendix is contained within the hernia sac. When the herniated appendix becomes inflamed, the clinical presentation may mimic an incarcerated or strangulated groin hernia, making preoperative diagnosis challenging. Computed tomography (CT) can facilitate accurate diagnosis by demonstrating the femoral location of the hernia and identifying the appendix within the hernia sac. We report an 84-year-old man who presented with a 4-day history of a painful, irreducible right groin swelling and vomiting, initially suspected clinically to represent an incarcerated right inguinal hernia. Contrast-enhanced CT demonstrated a right femoral hernia containing an inflamed appendix continuous with the cecum, enabling a preoperative diagnosis of De Garengeot hernia with acute appendicitis. The patient underwent appendicectomy and primary suture repair of the femoral hernia, with histopathological confirmation of acute appendicitis and serositis. This case highlights the importance of careful CT assessment of an irreducible groin mass. Recognition of the femoral location of the hernia and demonstration of continuity of its tubular content with the cecum are key imaging features that can facilitate accurate preoperative diagnosis and inform surgical management.

## Introduction

De Garengeot hernia, first described by René-Jacques Croissant de Garengeot in 1731, remains a rare and diagnostically challenging condition. Contemporary systematic review data demonstrate a marked female predominance and presentation predominantly in older adults. In a systematic review of 222 cases reported across 197 publications, Guenther et al. found that 81.1% of patients were female, with a mean age at presentation of 69.8 years [1]. Despite increasing availability of cross-sectional imaging, a specific preoperative diagnosis was established by imaging in only 31.5% of reported cases [1].

The clinical presentation is often nonspecific, most commonly consisting of a painful or tender groin swelling, and may resemble a conventional incarcerated or strangulated groin hernia [1,2]. This clinical overlap contributes to the diagnostic difficulty, with the majority of reported cases historically diagnosed at the time of surgery [1]. Accurate preoperative identification is nevertheless clinically relevant because knowledge of the hernia contents, the condition of the appendix, and the presence of associated complications can inform operative planning [[1], [2], [3]].

Computed tomography provides useful anatomical assessment for establishing the diagnosis preoperatively. Characteristic findings include a tubular structure situated within the femoral canal, typically ventral and medial to the femoral vein, which can be traced proximally to the cecum and terminates blindly [[3], [4], [5], [6]]. Multiplanar reformatted images are particularly useful for demonstrating this anatomical continuity [[3], [4], [5], [6]]. When associated appendicitis is present, appendiceal enlargement and surrounding inflammatory change may also be identified, while CT permits assessment for complications including perforation and abscess formation [[3], [4], [5], [6]].

We report a preoperatively diagnosed De Garengeot hernia with acute appendicitis in an 84-year-old man who presented with an irreducible painful right groin mass initially suspected clinically to represent an incarcerated inguinal hernia. The case differs from the predominant demographic reported in the literature and, more importantly, illustrates a specific preoperative CT diagnosis in a condition that remains frequently unrecognized before surgery. Recognition of the femoral location of the hernia and demonstration of continuity of its blind-ending tubular content with the cecum enabled the correct diagnosis and provided relevant information for subsequent surgical management.

## Case presentation

An 84-year-old man presented to the emergency department with a 4-day history of a painful right groin swelling, associated with early satiety and abdominal fullness. He vomited on the day of presentation but continued to open his bowels.

On examination, he was afebrile and hemodynamically stable. The abdomen was soft and nontender. A firm, tender, nonreducible right groin mass measuring approximately 8 × 6 cm was present. The initial clinical impression was an incarcerated right inguinal hernia, with strangulation requiring exclusion. Laboratory investigations demonstrated mild leukocytosis (white cell count 12.5 × 10⁹/L), a C-reactive protein level of 8.1 mg/L and a normal serum lactate of 0.8 mmol/L. Serum sodium was reduced at 125 mmol/L. No preoperative ultrasound was performed.

Contrast-enhanced CT of the abdomen and pelvis was performed on the day of presentation. This demonstrated a right femoral hernia, with the hernia neck situated inferior to the inguinal ligament and medial to the right femoral vein, causing mild compression of the adjacent femoral vein (Fig. 1). The hernia neck measured approximately 14 mm on sagittal reformatted images. The hernia sac contained a blind-ending tubular structure continuous with the cecum, representing the appendix extending into the femoral canal (Fig. 2). The appendix was enlarged, measuring approximately 12 mm in diameter, with mural thickening and enhancement, associated periappendiceal fluid, marked surrounding fat stranding and adjacent right groin soft-tissue edema (Fig. 3). No appendicolith was identified. There was no evidence of appendiceal perforation, abscess formation or extraluminal gas. No free intraperitoneal air or fluid or associated bowel obstruction was present. The CT appearances were diagnostic of a right De Garengeot hernia with acute appendicitis.

**Fig. 1:**
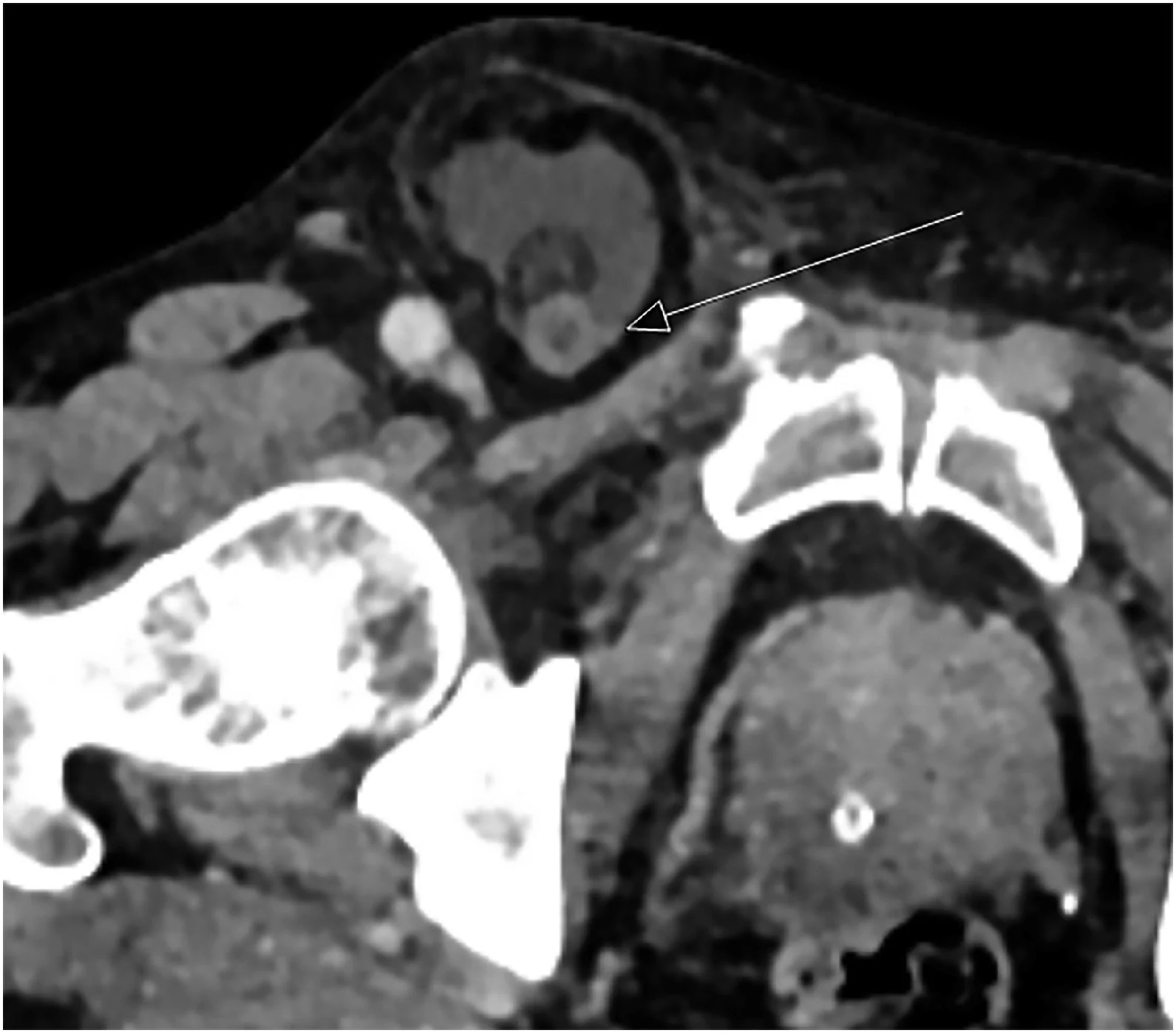
Axial contrast-enhanced CT image demonstrating the inflamed appendix within the right femoral hernia sac (arrow), with surrounding inflammatory fat stranding and fluid. The hernia lies medial to and compresses the adjacent right femoral vein, confirming its location within the femoral canal.

**Fig. 2:**
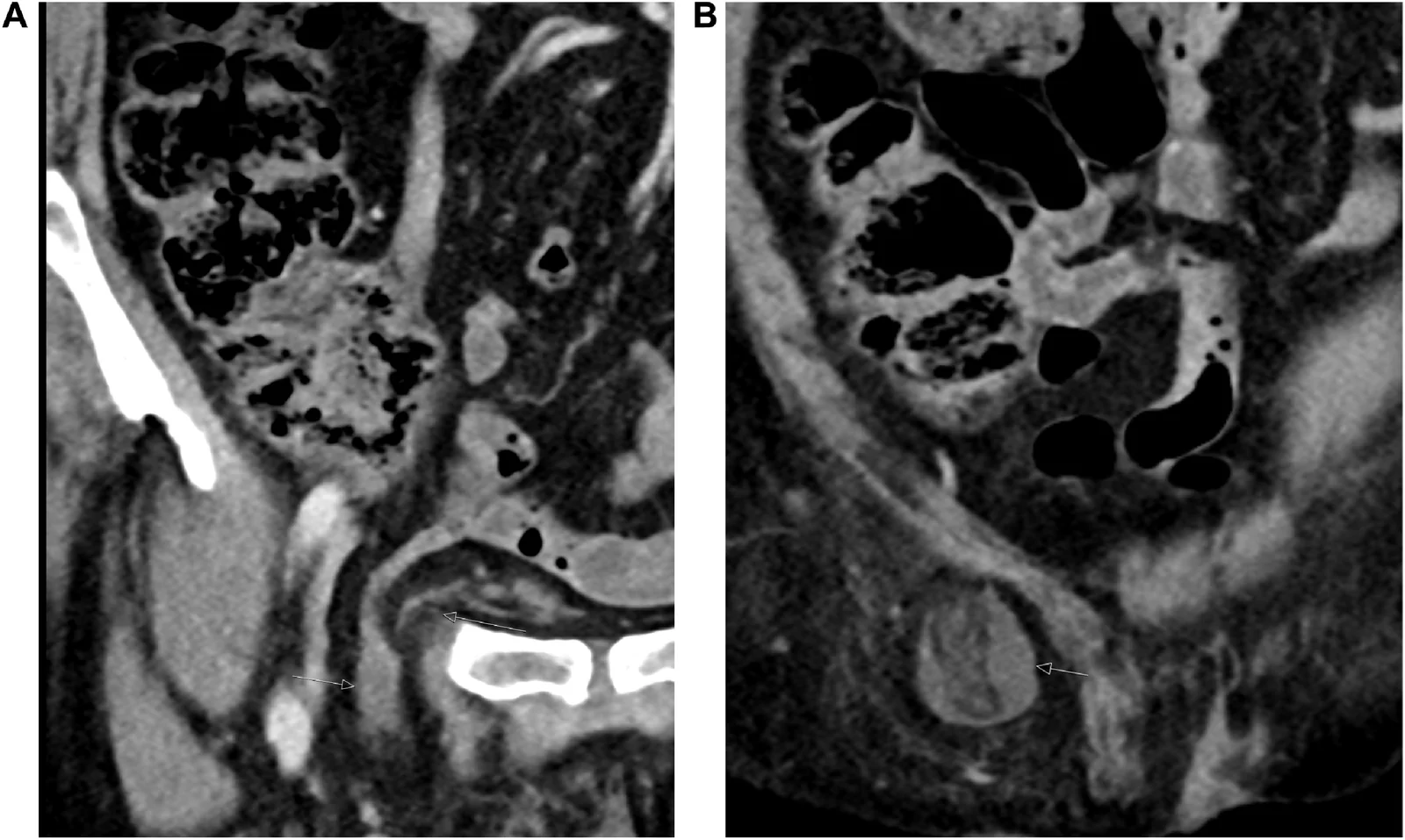
Coronal contrast-enhanced CT images demonstrating a right De Garengeot hernia with acute appendicitis. (A) The appendix extends into the right femoral canal; the upper arrow indicates the femoral canal and the lower arrow indicates the herniated appendix. (B) Periappendiceal fluid adjacent to the inflamed herniated appendix (arrow).

**Fig. 3:**
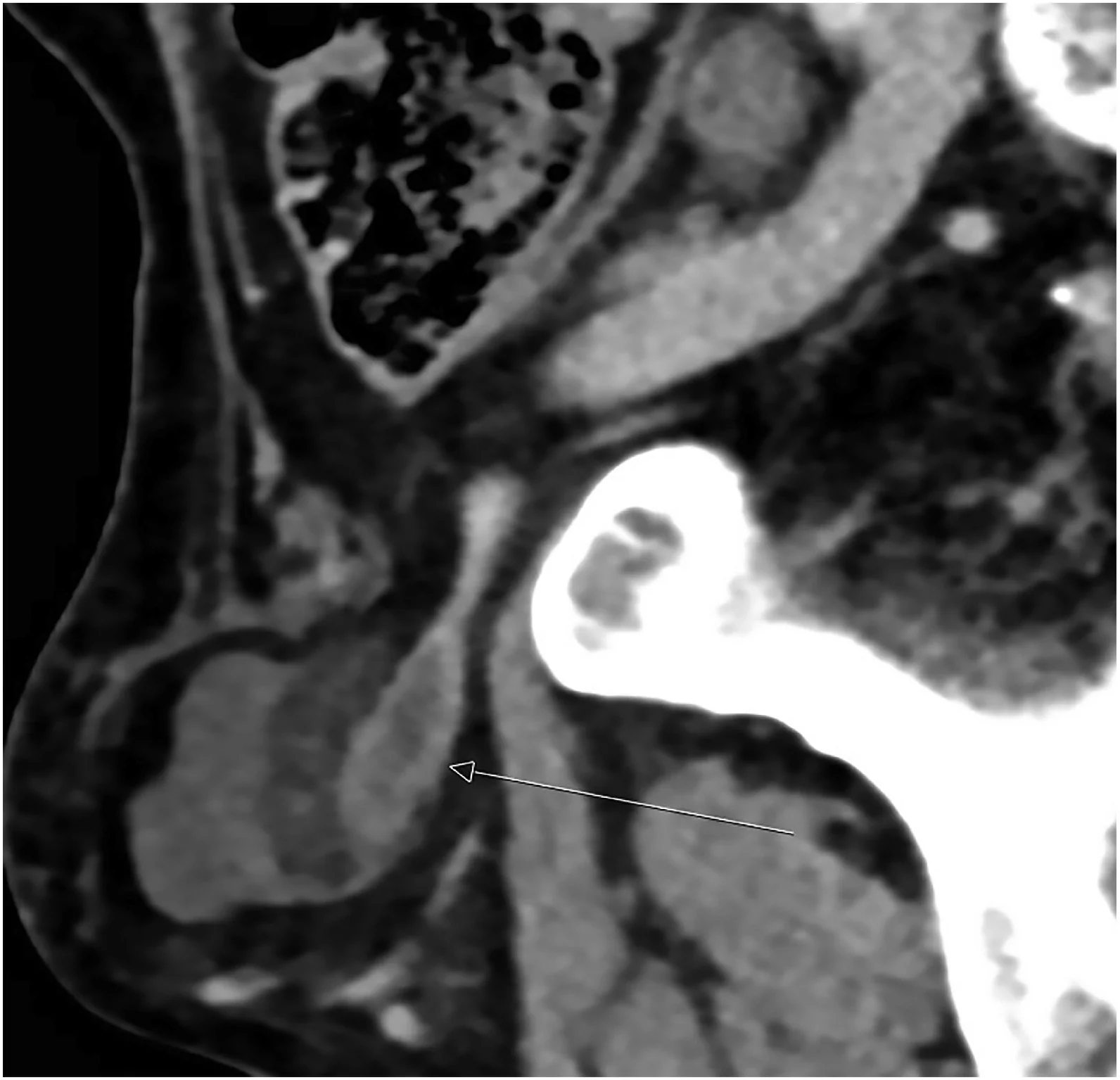
Sagittal contrast-enhanced CT image demonstrating the appendix (arrow) arising from the cecum and extending inferiorly towards the femoral hernia, with surrounding inflammatory changes and adjacent fluid.

Following CT, the patient was transferred for surgical management and underwent surgery the following day. A McEvedy approach was used, with operative findings confirming a right femoral hernia containing the appendix. Appendicectomy was performed, the hernia sac was reduced, and the femoral canal was repaired primarily with interrupted sutures without prosthetic mesh. There were no immediate operative complications.

Histopathological examination confirmed acute appendicitis with serositis, with no evidence of perforation, granulomatous inflammation, parasitic infection or neoplasia. The immediate postoperative surgical course was uncomplicated. The patient subsequently developed transient postoperative delirium and worsening of the hyponatremia present on admission, requiring medical management and prolonging his hospital stay. The surgical drain was subsequently removed with satisfactory wound healing. He recovered well and was discharged after approximately 30 days in hospital, with no further surgical follow-up required.

## Discussion

The present case demonstrates several recognized features of De Garengeot hernia while differing from the predominant demographic reported in the literature. In the systematic review by Guenther et al., which included 222 cases from 197 publications, 81.1% of patients were female and the mean age at presentation was 69.8 years [1]. Groin swelling and tenderness were the most frequently reported presenting features, occurring in 82.4% and 79.7% of cases, respectively [1]. Our patient was an 84-year-old man who presented with a painful, irreducible groin swelling, therefore differing from the marked female predominance while sharing the typical local presentation described in the literature. Preoperative diagnosis remains challenging: in the same systematic review, only 31.5% of cases were diagnosed by imaging before surgery [1]. The successful CT diagnosis in the present case is therefore noteworthy, particularly as the initial clinical impression was an incarcerated inguinal hernia.

CT is particularly valuable in establishing both the anatomical location of the hernia and the nature of its contents. Tsuruta et al. described useful CT features for recognizing De Garengeot hernia, including identification of a tubular structure ventral and medial to the femoral vein, continuity of this structure with the cecum and demonstration of its blind-ending configuration [3]. Multiplanar CT images facilitate demonstration of these anatomical relationships [[3], [4], [5], [6], [7]]. In our patient, the hernia neck was located inferior to the inguinal ligament and medial to the femoral vein, while multiplanar images demonstrated a blind-ending tubular structure continuous with the cecum, enabling confident identification of the appendix within the femoral canal. Associated appendiceal enlargement, mural enhancement, periappendiceal fluid and inflammatory fat stranding supported the diagnosis of acute appendicitis, while the absence of extraluminal gas or abscess indicated no CT evidence of perforation.

The differential diagnosis of an irreducible or inflammatory groin mass includes an incarcerated femoral or inguinal hernia containing bowel or omentum, inflammatory lymphadenopathy and groin abscess [4,7,8]. Amyand hernia is a particularly relevant differential because it also contains the vermiform appendix. The distinction is primarily anatomical: in De Garengeot hernia the appendix is contained within a femoral hernia, whereas in Amyand hernia the appendix lies within an inguinal hernia [7,8]. Careful assessment of the relationship of the hernia to the inguinal ligament and femoral vessels, together with multiplanar demonstration of the course and cecal continuity of the appendix, facilitates differentiation.

There is no universally accepted operative strategy for De Garengeot hernia, and management is influenced by the condition of the appendix and the presence of perforation or contamination [1,9]. Appendicectomy with femoral hernia repair is commonly performed when acute appendicitis is present, although different open and laparoscopic operative approaches have been described [1,9,10]. A systematic review by Gómez-Portilla et al. demonstrated that entirely laparoscopic management using transabdominal preperitoneal or total extraperitoneal techniques is feasible in selected patients, although published experience remains limited [10].

The method of hernia repair requires particular consideration when appendicitis is present. In the meta-analysis by Linder et al., mesh or plug repair was used in 22 patients and suture repair in 59 patients; mesh was not used in patients with perforated appendicitis [9]. The authors concluded that mesh material does not appear advisable in the presence of perforation [9]. The choice of prosthetic or primary repair should therefore take account of appendiceal perforation and the degree of operative contamination. In our patient, the appendix was inflamed but nonperforated, and appendicectomy with primary suture repair without prosthetic mesh was performed.

From a radiological perspective, the principal teaching point is that De Garengeot hernia may clinically resemble a more conventional incarcerated groin hernia and remain unrecognized before surgery [1,7,9]. Systematic evaluation of the anatomical location of the hernia, followed by careful assessment of its contents and their continuity with intra-abdominal structures, can permit a specific preoperative diagnosis [[3], [4], [5], [6], [7], [8]]. In the present case, accurate localization of the hernia to the femoral canal and demonstration of continuity of the herniated appendix with the cecum established the diagnosis before surgery and provided clinically relevant information for operative planning.

## Patient consent

Written informed consent was obtained from the patient for publication of this case report and accompanying images.
